# As COVID-19 cases surge despite mass vaccination, it’s time to focus on the vulnerable

**DOI:** 10.3332/ecancer.2021.ed117

**Published:** 2021-11-04

**Authors:** Martin Little, Matthew Fittall, Hayley McKenzie, Michael Tilby, Arvind Tripathy, Lennard YW Lee

**Affiliations:** 1 Oxford University Hospitals, Oxford, OX3 7LE, UK; 2 The Royal Marsden Hospital, London, SW3 6JJ, UK; 3 University Hospital Southampton, Southampton, SO16 6YD, UK; 4 University Hospitals Birmingham, Birmingham, B15 2GW, UK; 5 New Cross Hospital, Wolverhampton, WV10 0QP, UK; 6 University of Oxford, Oxford, OX3 7DQ, UK; * joint first author

**Keywords:** COVID-19, cancer, vaccination

## Abstract

The COVID-19 pandemic is an era-defining, international emergency impacting the global economy, politics and countless individual lives. People living with cancer have increased risk of hospitalisation and mortality from COVID-19. There are limited data regarding vaccine efficacy in people with cancer, with lack of empirical evidence to guide vaccine strategy in cancer patients fostering uncertainty. Vulnerable groups, for whom vaccination protection may be attenuated, now carry the greatest burden of risk amongst the population. The cancer community needs to reconsider the potential on-going impact of COVID-19 and develop and plan new programs of work to mitigate it. Multiple potential future scenarios now exist, ranging from full protection from COVID-19 for cancer patients via herd immunity to viral evolution for vaccine resistance and increased virulence. Defining those most vulnerable to COVID-19 post-vaccination will require large-scale data and evidence to comprehensively identify factors that reduce vaccine efficacy. Once identified, protecting these groups through transmission and mortality risk reduction will become paramount. As the pandemic progresses, “protecting the vulnerable” may enable a return to normal for the majority, whilst still protecting individuals living with and beyond cancer who already live with the challenges of having a cancer diagnosis.

The COVID-19 pandemic is an era-defining, international emergency impacting the global economy, politics and countless individual lives. International infection and mortality rates remain considerably more than 18 months into the pandemic.

People living with cancer have increased risk of hospitalisation and mortality from COVID-19 [1, 2]. Treatment with systemic anticancer therapy and radiotherapy does not appear to impact on outcomes of infection [3]. However, these data were collected prior to widespread vaccine.

COVID-19 vaccination is clearly effective in the general population, with a wealth of trial evidence and real-world data to support this [4-6]. By contrast, there are limited data regarding vaccine efficacy in people with cancer. Further antibody and T-cell responses to COVID-19 vaccination has only been assessed in small cohorts of patients, but generally show evidence of attenuated responses [7, 8]. Similarly, safety data specific to the oncology population is lacking, particularly with respect to the rare haematological complications [9]. The lack of empirical evidence to guide a vaccine strategy in cancer patients has created uncertainty. The impact of the timing of vaccination alongside the broad array of systemic anticancer therapies (SACT) in unknown. National guidelines for combining COVID-19 vaccination with SACT are extrapolated from experience with other infections, again based on limited evidence [10, 11]. The threat that new variants pose, with increased transmissibility, mortality and vaccine-resistance, is only likely to be greater in the cancer population for whom vaccination efficacy is less certain.

The United Kingdom was one of the first countries to achieve >80% first-dose coverage of the COVID-19 vaccination in its adult population. As new infection numbers rise rapidly, it is becoming clear that mass national vaccination is not sufficient to completely suppress transmission. The current surge in cases is occurring principally in the incompletely vaccinated population, but some infections have occurred despite adequate vaccination.

It is essential to re-evaluate how mass vaccination has shifted the landscape of health and economic risks from COVID-19. Vulnerable groups for whom vaccination protection may be attenuated may now carry the greatest burden of risk amongst the population. Those living with and beyond cancer are likely to be foremost amongst the vulnerable. The cancer community needs to reconsider the potential on-going impact of COVID-19 and develop and plan new programs of work to mitigate it.

The ideal scenario is that the 2021 mass national vaccination program leads to protection from hospitalisation in most individuals. As the pool of unvaccinated people dwindles, cases will rapidly peak and COVID-19 might rapidly fade into medical history. Potentially, the current surge amongst the unvaccinated might have minimal or no effect on patients living with and beyond cancer.

Two other scenarios need to be considered which would have differential impact on the general and cancer population. An intermediate scenario assumes that vaccine efficacy is not complete and/or reduces with time. The wider population may be protected; however, there may be a vulnerable minority group that remain at heightened risk of hospitalisation and death from COVID-19. Cancer patients are likely to be a large constituent of this vulnerable group because of reduced vaccine efficacy due to the immunosuppressive effects of chemotherapy or the cancer. In this scenario, the current exponential rise in cases would occur initially in the unvaccinated but reach much higher levels than previous waves. This effect may be exacerbated due to belief that vaccination has been fully efficacious and adherence to other COVID-19 measures such as social distancing, PPE, contact tracing, hand washing and mass testing are reduced. As cases surge, outbreaks and deaths may start to be observed in cancer patients. In this scenario, the ongoing impact of COVID-19 will be focussed on vulnerable groups, with minimal impact on the wider population who remain protected from hospitalisation/death.

In a final worst-case scenario, vaccine efficacy may be significantly reduced or neutralised by viral evolution or time. Routine viral sequencing, at a scale never seen before, has detailed the ongoing evolution of the SARS-CoV-2. There is evidence that the current dominant variant, the delta variant (B.1.617.2), has increased transmissibility and pathogenicity [12]. In a number of countries, COVID-19 re-infection is common [13]. In this scenario, both the wider population and cancer patients will be at equal risk of being infected by COVID-19. Hospitalisation and mortality will be rise in both groups, albeit at a higher level amongst cancer patients as seen in the pre-vaccination period.

It is hoped that vaccination is successful at reducing the greatest burden of COVID-19 morbidity and mortality, which has been seen in the elderly. Prior to vaccination, deprivation of liberty and legally enforced social distancing were the only measures to protect healthcare services (“protect the NHS”) and maintain confidence in the safety net that they provide. The emphasis has rightly been on establishing the efficacy of vaccination in the general and elderly population. In the next phase, where the aim is to minimise death and hospitalisation whilst maximising social and economic recovery, a shift towards a strategy to “protect the vulnerable” might be required. This requires us to both accurately define those that remain most vulnerable post-vaccination, and what measures can be enacted to protect them.

In addition to national benefits of a focused COVID-19 vaccination, the wider issue of international infection rates in low- and middle-income countries (LMICs) needs to be considered. High-income countries such as the UK have secured the majority of the global vaccine supply for their own populations, yet highly transmissible and resistant variants have arisen in countries such as India and Brazil. Limiting third vaccinations to specific, vulnerable populations in high-income countries would allow more vaccines to be delivered globally. This would align with the aims of COVAX, a joint venture between the World Health Organisation, CEPI, Gavi and UNICEF to provide COVID-19 vaccines to LMICs.

Defining those most vulnerable to COVID-19, despite vaccination, will require large-scale data and evidence to comprehensively identify factors that reduce vaccine efficacy. National datasets could provide the scale required for the highest granularity and the infrastructure required for real-time analysis. Clearly not all those with a diagnosis of cancer will be at the same risk. It is anticipated that the haematological malignancies and the most myelosuppressive therapies will pose the greatest risk. It is likely that many with more limited stage solid organ malignancies will carry only a background population risk, empowering them to follow the less restrictive measures enjoyed by the rest of the population.

Once the vulnerable are defined, all levers must be utilised to protect them. This might focus on two priorities: minimising risk of transmission and reducing mortality from COVID-19. Minimising transmission might require varied approaches, including easier access to COVID-19 testing for friends and family, targeted education/engagement, maintained social distancing, resources to further reduce the risk of COVID-19 from hospital and prioritised access to any COVID-19 booster programs. In terms of reducing mortality, national rapid trials in the vulnerable should be funded to implement and test new therapeutics in these vulnerable groups.

## Conclusion

There will be significant benefits from a COVID-19 strategy that “protects the vulnerable.” However, it is important to consider the potential limitations. To date, the bulk of the COVID-19 responses have focused on population level approaches, with COVID-19 treatment and vaccination studies principally recruiting individuals who do not have cancer and may not be vulnerable. A very careful and co-ordinated shift in terms of the mindset of the public health, clinical, academic and research stakeholders may require resource re-allocation. Efficient resource allocation can reduce the financial impact of the pandemic. Furthermore, it would enable the COVID-19 response to be tailored to the individuals and facilitate a shift away from the mass interventions, where the scale of benefit for each individual is minimal. As the pandemic progresses, “protecting the vulnerable” may enable a return to normal for the majority, whilst still protecting individuals living with and beyond cancer who already live with the challenges of having a cancer diagnosis.

## Conflicts of interest

Martin Little – Pfizer, payment for lecture.

The remaining authors declare no conflicts of interest.

## Funding statement

None.
